# Gut–brain axis mechanisms of remote brain dysfunction after traumatic spinal cord injury: immune inflammation, the vagus nerve, and neuroendocrine pathways

**DOI:** 10.3389/fncel.2026.1879641

**Published:** 2026-07-15

**Authors:** Yutong Liu, Haifeng Zhang, Bingkai Ren

**Affiliations:** 1 Affiliated Hospital of Jiangxi University of Chinese Medicine, Nanchang, China; 2 Jiangxi University of Chinese Medicine, Nanchang, China

**Keywords:** gut–brain axis, gut-derived pathological signals, neuroendocrine dysregulation, neuroinflammation, remote brain dysfunction, spinal cord injury, vagus nerve

## Abstract

Traumatic spinal cord injury (SCI) has traditionally been regarded as a central nervous system injury mainly confined to the injured segment. However, increasing evidence indicates that SCI can also induce neuroinflammation, cognitive decline, and emotional disorders in remote brain regions, suggesting that its pathological impact is systemic rather than purely local. Remote brain dysfunction after SCI is unlikely to be driven by a single pathway, but may arise from the combined effects of systemic inflammation, autonomic imbalance, neuroendocrine dysregulation, and disruption of intestinal homeostasis. Unlike previous reviews that mainly discuss SCI-associated gut dysbiosis, neuroinflammation, or gut–brain communication separately, this review organizes SCI-related intestinal abnormalities around the concept of “gut-derived pathological signals” and further distinguishes direct SCI evidence from cross-disease mechanistic evidence and proposed mechanistic inference. Specifically, we summarize how intestinal dysmotility, microbial metabolic remodeling, abnormalities in short-chain fatty acids (SCFAs), barrier vulnerability, and mucosal immune imbalance after SCI may generate persistent pathological signals. We then analyze how these signals may affect the central nervous system through an immune-inflammatory main axis, a vagal neural relay branch, and a neuroendocrine modulatory branch, ultimately converging on a common downstream brain effector stage characterized by blood–brain barrier impairment, neuroinflammation, synaptic plasticity deficits, and dysfunction of key brain regions such as the hippocampus and medial prefrontal cortex (mPFC). Based on this cascade, we propose a stratified intervention framework involving upstream restoration of intestinal homeostasis, midstream regulation of interorgan transmission pathways, and downstream protection of brain effector mechanisms. Overall, this review provides an evidence-stratified gut–brain axis framework for understanding remote brain dysfunction after SCI and highlights the need for SCI-specific temporal mapping and pathway-selective causal validation.

## 1 Remote brain dysfunction after SCI and the gut–brain axis perspective

Traumatic spinal cord injury (SCI) has traditionally been understood as a central nervous system injury mainly confined to the injured segment. The classic pathological framework emphasizes primary mechanical injury and the subsequent secondary injury cascade, including ischemia and hypoxia, disruption of ionic homeostasis, excitotoxicity, oxidative stress, and amplification of local inflammation (Ahuja et al., 2017; David and Kroner, 2011; Shultz and Zhong, 2017). This framework can relatively well explain the loss of neurons and glial cells within the injured spinal cord segment, as well as the occurrence of motor and sensory deficits. However, it remains insufficient to explain systemic comorbid phenotypes after SCI, such as neuroinflammation in remote brain regions, cognitive decline, and emotional disorders.

In recent years, increasing evidence has shown that even in the absence of direct brain injury, SCI can induce neuroinflammatory responses and functional alterations in remote brain regions (Faden et al., 2016). Clinically, the prevalence of depression is markedly increased after SCI, and the long-term risks of chronic neurological, psychiatric, and endocrine comorbidities are also elevated (Williams and Murray, 2015; Mashlah et al., 2025). Cognitive impairment is likewise increasingly recognized as an important component of long-term outcomes after SCI, mainly manifesting as impairments in attention, executive function, memory, and information processing speed (Molina et al., 2018; Portela Hara et al., 2024; Sachdeva et al., 2018; Wu et al., 2014). Experimental studies have further demonstrated that SCI can lead to persistent brain inflammation and neurodegenerative changes, while interventions targeting microglia-mediated inflammatory processes can alleviate chronic brain pathology and improve recovery outcomes (Li et al., 2020). Recent evidence also shows the presence of remote neuroinflammation in the prefrontal cortex after SCI, suggesting that SCI-related brain pathology is not limited to classic vulnerable regions such as the hippocampus (Ning et al., 2026). Overall, these findings indicate that brain dysfunction after SCI is not a delayed extension of local spinal cord pathology, but is more likely to have a progressive systemic pathological basis.

Mechanistically, remote brain dysfunction after SCI is unlikely to depend on a single pathway, but may be jointly driven by multiple interorgan pathological processes. Persistent systemic inflammation and peripheral immune reprogramming after injury may provide a long-term background for innate immune activation in the brain. Meanwhile, autonomic imbalance, abnormal neuroendocrine stress responses, and alterations in visceral sensory inputs such as vagal afferent signaling may continuously affect central regulatory networks. At the same time, SCI is often accompanied by intestinal dysmotility, gut microbial dysbiosis, barrier disruption, and impaired mucosal immune homeostasis. These changes not only constitute the basis of local gastrointestinal complications, but may also transmit abnormal peripheral information to the brain through humoral, neural, and neuroendocrine pathways (Schmidt et al., 2021; Besecker et al., 2020; Valido et al., 2022; Kigerl et al., 2020; Prüss et al., 2017). Therefore, remote brain dysfunction after SCI should be understood more as the result of multiple interacting interorgan pathological processes rather than as the linear extension of a single pathological chain. Recent psychoneuroimmunoendocrine studies in SCI also support this view from the perspective of systemic pathology (Ortega et al., 2023).

Among these potential mechanisms, the gut–brain axis provides a particularly integrative framework. Its significance lies not in excluding other mechanisms, but in linking SCI-induced autonomic and neuroendocrine imbalance, intestinal dysmotility, microbial metabolic remodeling, barrier vulnerability, and mucosal immune disturbance into a continuous pathological cascade. In other words, after SCI, the gut may not merely be a passively affected organ, but may gradually evolve into a peripheral pathological source that continuously generates abnormal signals. These gut-derived pathological signals can then act on the brain through immune-inflammatory, vagal, and neuroendocrine pathways, ultimately converging on a common downstream brain effector stage characterized by activation of neuroinflammatory networks, impaired synaptic plasticity, and dysfunction of key brain regions such as the hippocampus and medial prefrontal cortex (Wu et al., 2014; Schmidt et al., 2021; Besecker et al., 2020; Valido et al., 2022; Kigerl et al., 2020; Prüss et al., 2017; Keane et al., 2025; Krishnapriya et al., 2025). From this perspective, the gut–brain axis provides an integrative framework for explaining the systemic, delayed, and persistent features of remote brain dysfunction after SCI.

Previous reviews have mainly focused on individual components such as SCI-induced gut dysbiosis, neuroinflammation, autonomic dysfunction, or psychological comorbidities. In contrast, the novelty of this review lies in integrating intestinal dysmotility, microbial metabolic remodeling, barrier vulnerability, and mucosal immune imbalance into the concept of gut-derived pathological signals. We further organize these signals into three evidence-stratified routes—an immune-inflammatory main axis, a vagal neural relay branch, and a neuroendocrine modulatory branch—to explain how intestinal abnormalities after SCI may contribute to remote hippocampal and mPFC dysfunction.

**Figure 1:**
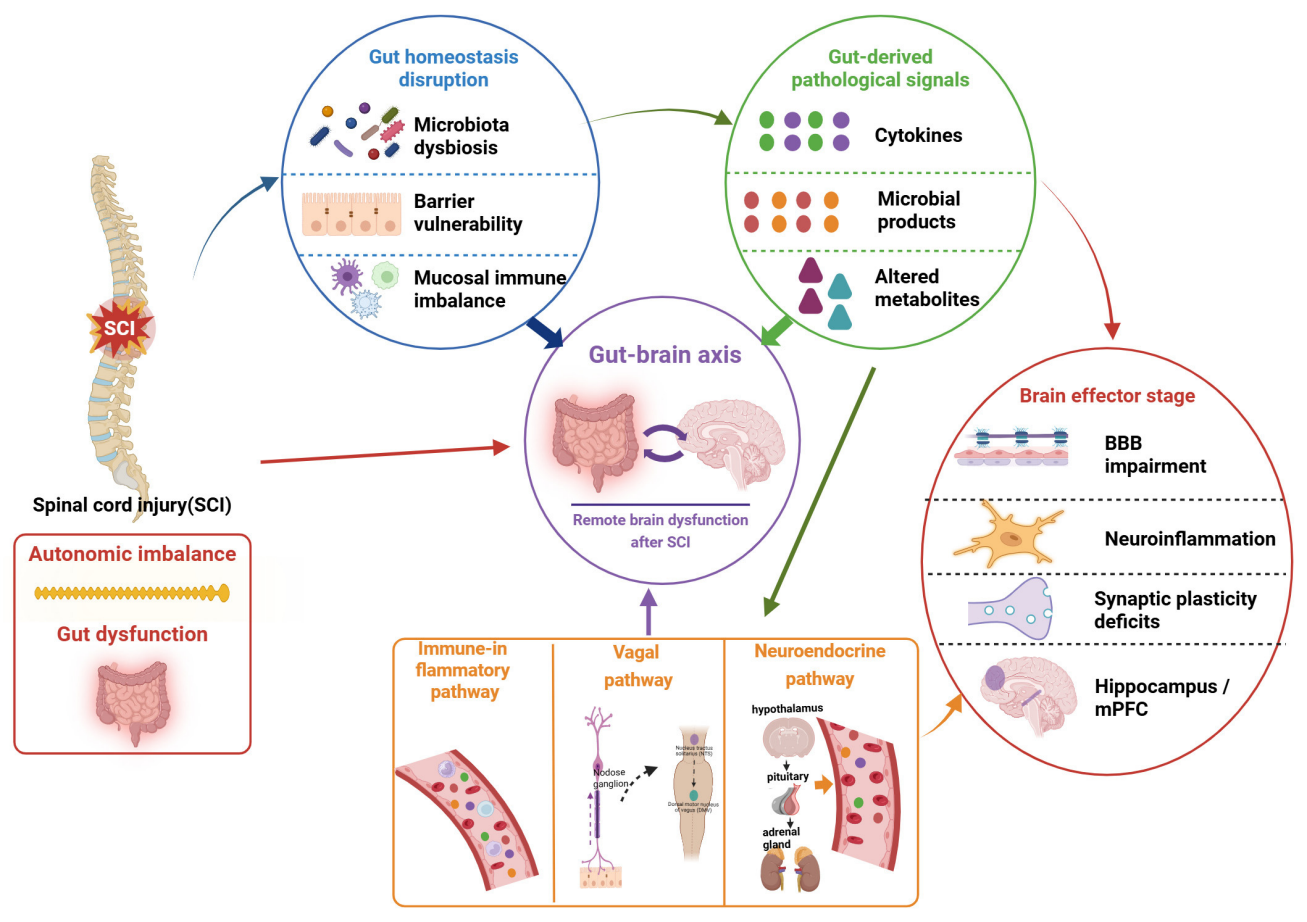
Conceptual framework of SCI-associated gut–brain axis dysregulation. SCI disrupts intestinal homeostasis through autonomic imbalance, gut dysfunction, microbiota dysbiosis, barrier vulnerability, and mucosal immune imbalance. These changes promote the formation of gut-derived pathological signals, including cytokines, microbial products, and altered metabolites. These signals may act on remote brain regions through immune-inflammatory, vagal, and neuroendocrine pathways, ultimately converging on a common downstream brain effector stage characterized by blood–brain barrier (BBB) impairment, neuroinflammation, synaptic plasticity deficits, and hippocampal/mPFC dysfunction. SCI, spinal cord injury; mPFC, medial prefrontal cortex.

This conceptual framework is summarized in Figure 1.

Therefore, this review centers on “gut-derived pathological signals.” First, we discuss how SCI gradually generates persistent abnormal peripheral inputs through autonomic and neuroendocrine imbalance, intestinal dysmotility, microbial metabolic remodeling, barrier vulnerability, and mucosal immune disturbance. Subsequently, we analyze how these signals act on the central nervous system through immune-inflammatory, vagal, and neuroendocrine pathways. Finally, based on this pathological cascade, we summarize stratified intervention strategies and translational prospects.

## 2 SCI-induced remodeling of intestinal homeostasis and the formation of gut-derived pathological signals

### 2.1 Autonomic and neuroendocrine imbalance after SCI: the initial link in disruption of intestinal homeostasis

Spinal cord injury (SCI) not only interrupts local neural conduction at the injured segment, but also profoundly disturbs the autonomic and neuroendocrine regulation of peripheral organs by the central nervous system. Intestinal homeostasis is not maintained solely by the enteric nervous system, but is jointly coordinated by parasympathetic vagal input, spinal sympathetic pathways, and local neural networks. Under physiological conditions, this multilevel regulatory system maintains gastrointestinal motility, mucosal secretion, local blood flow, epithelial barrier integrity, and immune homeostasis within gut-associated lymphoid tissue (GALT), thereby providing a relatively stable host environment for the gut microbiota. Therefore, the pathological significance of intestinal abnormalities after SCI should not be simplified as local complications such as bowel dysfunction, but should be understood as an important link in the systemic pathological expansion of central injury to peripheral organs (Kigerl et al., 2020).

At the autonomic level, SCI, especially injury at the thoracic level or above, can interrupt the descending control of spinal autonomic networks by the brainstem and supraspinal centers, resulting in impaired sympathetic output and disruption of the existing sympathetic–parasympathetic balance in the gut. Clinical studies show that patients with SCI can develop definite neurogenic bowel dysfunction, whose clinical and pathophysiological features are closely related to the injury pattern. Symptoms such as abdominal distension, constipation, and defecation disorders suggest persistent impairment of intestinal propulsion and emptying after injury (Vallès et al., 2006). Animal studies further indicate that chronic high-level SCI can prolong total gastrointestinal transit time, reduce fecal output, and be accompanied by decreased cholinergic responsiveness of colonic smooth muscle, suggesting that SCI can directly impair intestinal propulsive function (Frias et al., 2019). Therefore, the consequences of autonomic imbalance after SCI are not limited to impaired motor function itself, but may also extend to mucosal secretion, local blood flow, and luminal micro-niches, thereby promoting remodeling of the intestinal microenvironment.

In addition to impaired sympathetic output, vagal regulation may also be indirectly affected after SCI. Although vagal fibers do not reach the gastrointestinal tract via the spinal cord, experimental studies show that high-level SCI can induce dysfunction of gastric vagal afferents. This suggests that SCI may indirectly affect vagal sensory–motor circuits by altering the local gastrointestinal signaling environment and brainstem integration (Besecker et al., 2020). Therefore, SCI-induced autonomic abnormalities should not be understood only as reduced sympathetic innervation, but may also involve functional changes at the vagal sensory interface. This indicates that disruption of intestinal homeostasis after SCI may not only affect motility and secretion, but may also alter the local environment sensed by vagal afferents.

Meanwhile, as a severe traumatic event, SCI can also induce a persistent neuroendocrine stress response. Experimental evidence indicates that SCI can induce abnormal sympathetic–neuroendocrine–adrenal reflexes, exposing the periphery long-term to dysregulated levels of glucocorticoids and catecholamines, thereby remodeling immune homeostasis (Prüss et al., 2017). The actions of these mediators may not be limited to immune organs, but may also involve the intestinal mucosal microenvironment. Evidence from studies of the intestinal barrier suggests that abnormal patterns of glucocorticoid and catecholamine exposure may participate in intestinal barrier remodeling by affecting epithelial renewal, mucus secretion, local immune tolerance, and regulation of tight junctions. However, in the context of SCI, this pathway should currently still be regarded as a mechanistically plausible inference, because the complete chain from neuroendocrine abnormalities to intestinal barrier disruption still lacks direct validation (Prüss et al., 2017; Boivin et al., 2007).

From the perspective of the overall pathological process, intestinal remodeling after SCI should not be understood as a single time-point event, but rather as a temporally evolving process. In the early phase after injury, disruption of intestinal homeostasis may be initiated mainly by autonomic imbalance and neuroendocrine reprogramming, leading to impaired intestinal motility, altered mucosal secretion, local microenvironmental stress, and early barrier vulnerability. During the subacute phase, persistent dysmotility and changes in the mucosal milieu may further reshape the microbial niche, promote microbial compositional and metabolic remodeling, and contribute to reduced production of short-chain fatty acids (SCFAs), especially butyrate. In the chronic phase, sustained gut dysbiosis, impaired barrier regulation, bacterial translocation, and low-grade systemic inflammation may interact with each other and maintain continuous gut-derived pathological signaling. Therefore, SCI-induced intestinal abnormalities should be viewed as a dynamic cascade progressing from neural and neuroendocrine dysregulation to microbial metabolic remodeling, barrier vulnerability, mucosal immune deviation, and amplification of gut-derived inflammation (Besecker et al., 2020; Prüss et al., 2017; Vallès et al., 2006; Frias et al., 2019; Boivin et al., 2007; Diaz et al., 2021; Kigerl et al., 2016).

It should also be emphasized that the extent and temporal pattern of intestinal remodeling after SCI are unlikely to be uniform across all patients or models. Rather, they may be influenced by injury level, injury severity, completeness of injury, the degree of autonomic disruption, bowel management, diet, infection, medication exposure, and other clinical or experimental conditions. Therefore, although thoracic or higher-level SCI is more likely to disturb autonomic control of the gut, it should not be assumed that all thoracic SCI cases lead to the same degree or trajectory of intestinal remodeling. Future studies should establish SCI-specific temporal maps of intestinal dysbiosis, barrier dysfunction, microbial metabolite alterations, and systemic inflammatory activation, and should further clarify how these changes vary according to injury characteristics and clinical context.

### 2.2 Intestinal dysmotility and microbial metabolic remodeling: from dysbiosis to abnormalities in SCFAs

Against the background of autonomic and neuroendocrine imbalance, one of the most direct functional consequences in the gut after SCI is delayed colonic transit and decreased emptying efficiency. For the gut, dysmotility is not merely delayed defecation; it also alters the retention time of luminal substrates, the local physicochemical environment, and exposure to bile acids and fermentable substrates, thereby remodeling the gut microbial niche. Previous studies have shown that gastrointestinal transit time itself can influence the composition of the gut microbiota. In addition, fecal levels of short-chain fatty acids (SCFAs) are significantly correlated with colonic transit time, and accelerated intestinal transit can increase butyrate concentrations in the distal colon. Therefore, in the context of SCI, intestinal dysmotility is not only a manifestation of neurogenic bowel dysfunction, but also a physical driving factor for subsequent microbial metabolic remodeling (Kashyap et al., 2013; Waseem et al., 2023).

Therefore, changes in the gut microbiota after SCI may progress from compositional alterations to functional disturbance. A study published in the Journal of Experimental Medicine showed that gut dysbiosis after SCI can persist and is associated with recovery outcomes and changes in gut-associated immune status. Clinical studies have also confirmed significant differences in the structure of the gut microbiota between patients with SCI and healthy controls. Among these, changes in microbial composition associated with bowel dysfunction and reductions in butyrate-producing bacteria appear to be relatively consistent findings. Decreased microbial diversity and specific microbial restructuring have also been observed in male patients with chronic complete traumatic SCI. These results suggest that gut microbial abnormalities after SCI are not simply manifested as a reduction in beneficial bacteria, but are more likely to reflect a shift in microbial function from supporting homeostasis toward a pro-inflammatory configuration (Kigerl et al., 2016; Gungor et al., 2016; Zhang et al., 2018).

One of the most important consequences of microbial functional remodeling is decreased production of SCFAs, especially reduced butyrate production. SCFAs are not only the main metabolic end products of dietary fiber fermentation by the gut microbiota, but also important mediators linking changes in microbial composition with host immune homeostasis. The classic study by Smith et al. demonstrated that SCFAs can regulate the size and function of the colonic Treg cell pool, thereby maintaining mucosal immune homeostasis. Park et al. further showed that SCFAs can influence the differentiation of effector T cells and regulatory T cells according to the local immune environment, suggesting their context-dependent regulatory effects on immune tolerance and inflammatory responses. Therefore, against the background of gut dysbiosis after SCI, decreased levels of SCFAs are not merely a reduction in microbial metabolites, but also reflect a weakened capacity of the gut to maintain local immune homeostasis and anti-inflammatory regulation (Smith et al., 2013; Park et al., 2015).

In addition to immune regulation, butyrate is an important energy substrate for colonic epithelial cells and directly participates in barrier homeostasis. Experimental studies show that butyrate can enhance intestinal epithelial barrier function by upregulating tight junction-related proteins. Recent mechanistic studies further suggest that n-butyrate regulates claudin-23 expression through the SP1/AMPK pathway, thereby participating in barrier homeostasis. Therefore, if intestinal dysmotility and microbial restructuring after SCI lead to reduced butyrate production, the pathological consequences may be dual: weakened immune tolerance and local anti-inflammatory regulation, together with impaired maintenance of the epithelial barrier, making the gut more prone to increased permeability and spillover of inflammatory signals. Specifically, abnormalities in SCFAs should not be regarded merely as secondary by-products of microbial changes, but may be key metabolic nodes linking intestinal dysmotility, mucosal immune deviation, and barrier vulnerability (Wang et al., 2012; Xu et al., 2023).

Recent SCI studies have begun to reconceptualize SCFAs from concomitant metabolites as pathological mediators with functional significance. A 2023 study showed that changes related to the gut microbiota and SCFAs in animal models of SCI may partially affect neurological functional recovery, suggesting that SCFAs may participate in broader gut–brain axis pathology after SCI. Therefore, decreased intestinal motility, microbial restructuring, and depletion of SCFAs after SCI should not be regarded as isolated phenomena. Instead, they constitute a continuous pathological process that drives the gut to transition from an organ supporting homeostasis to an interface that is prone to inflammation and capable of generating abnormal signals. Together, these changes provide a direct basis for the barrier vulnerability and mucosal immune imbalance discussed in the next section (Kigerl et al., 2016; Gungor et al., 2016; Zhang et al., 2018; Wang et al., 2012; Xu et al., 2023; Jing et al., 2023).

### 2.3 Barrier vulnerability and mucosal immune imbalance: formation of gut-derived pathological signals

After intestinal dysmotility, changes in microbial composition, and deficiency of SCFAs, the next key stage in the intestinal pathological process after SCI is the gradual formation of barrier vulnerability and mucosal immune imbalance. Animal studies indicate that SCI can increase intestinal permeability and promote bacterial translocation; rat models further show enhanced intestinal inflammatory responses after injury. Clinical studies likewise suggest that patients with chronic SCI have disruption of intestinal homeostasis, bacterial translocation, and activation of systemic inflammation. Meanwhile, SCI-related gut dysbiosis is associated with endotoxemia and amplification of local and systemic inflammation. Overall, these findings indicate that intestinal abnormalities after SCI are not limited to delayed transit or changes in microbial composition, but may gradually transform the gut from an interface that maintains host–microbial homeostasis into a pathological interface characterized by barrier vulnerability, mucosal susceptibility, and continuous generation of abnormal peripheral signals (Diaz et al., 2021; Kigerl et al., 2016; O'Connor et al., 2018; Myers et al., 2019).

Against this background, microbe-associated molecular patterns are more likely to be abnormally exposed to the intestinal mucosal immune system. Previous studies have shown that lipopolysaccharide (LPS) can activate Toll-like receptor 4 (TLR4) signaling in intestinal epithelial cells, providing a mechanistic basis for enhanced local innate immune activation after barrier disruption. In the context of SCI, recent studies further suggest that gut microbiota disturbance may be accompanied by activation of the TLR4/MyD88 pathway, supporting the view that barrier dysfunction is not only a result of epithelial injury, but may also promote amplification of innate immunity. However, this interpretation requires caution. Rather than assuming that the complete LPS/TLR4/NF-κB cascade after SCI has been fully confirmed, it is more appropriate to state that barrier vulnerability creates conditions for abnormal host exposure to microbial inflammatory signals and may thereby promote the continuous escalation of local inflammatory responses (Hornef et al., 2002; Rong et al., 2021).

Persistent mucosal inflammatory stress may also shift the intestinal immune environment from a tolerant state toward a more pro-inflammatory state. Classic immunological studies show that in an IL-6-rich inflammatory microenvironment, TGF-β promotes differentiation of pathogenic Th17 cells while inhibiting induction of Foxp3-positive regulatory T cells (Tregs). This provides an important mechanistic framework for understanding how chronic intestinal inflammation weakens mucosal immune tolerance. In the context of SCI, these findings suggest that once intestinal homeostasis is disrupted, the mucosal immune environment may gradually shift toward inflammatory polarization. However, direct quantitative evidence of local Th17/Treg imbalance in the intestinal mucosa after SCI remains limited. Therefore, this should be defined as a biologically plausible and mechanistically supported explanation rather than an established fact fully confirmed in SCI (Bettelli et al., 2006).

From the perspective of pathological progression, barrier vulnerability and mucosal immune imbalance are not isolated processes, but may form a mutually reinforcing vicious cycle. Barrier disruption increases abnormal exposure of the mucosal immune system to microbial products, while persistent inflammatory activation further weakens epithelial and immune homeostatic capacity. At this stage, the pathological significance of the gut after SCI begins to extend beyond local bowel dysfunction. The gut gradually evolves into a peripheral pathological source capable of continuously releasing abnormal signals, and may affect remote organs, especially the brain. In this sense, the formation of gut-derived pathological signals is a key turning point linking disruption of intestinal homeostasis with subsequent interorgan pathogenic mechanisms, and provides a common upstream basis for the three bridging pathways discussed in the next chapter (Diaz et al., 2021; Kigerl et al., 2016; O'Connor et al., 2018; Myers et al., 2019).

In summary, these findings indicate that the pathological significance of the gut after SCI is no longer limited to local bowel dysfunction, but lies in its role as a peripheral source of continuous abnormal signal generation. These abnormal signals include not only increased pro-inflammatory mediators and microbe-associated molecular patterns, but may also include intestinal sensory inputs that are abnormally remodeled in the context of barrier disruption, gut dysbiosis, and local inflammation (Besecker et al., 2020; Diaz et al., 2021; Kigerl et al., 2016). Meanwhile, neuroendocrine regulatory signals that are chronically amplified under persistent stress may also help maintain this abnormal peripheral signaling state (Prüss et al., 2017). Therefore, the gut after SCI should not be regarded merely as an injured organ, but should be viewed as a common upstream source of subsequent interorgan pathogenic mechanisms. This view lays the foundation for the next chapter, namely, a discussion of how gut-derived pathological signals act on the central nervous system through immune-inflammatory, vagal, and neuroendocrine pathways, ultimately promoting the occurrence and progression of remote brain dysfunction.

## 3 Three key pathways by which gut-derived pathological signals mediate remote brain dysfunction

In Section 2, we discussed how disruption of intestinal homeostasis after SCI gradually generates persistent gut-derived pathological signals through autonomic and neuroendocrine imbalance, microbial metabolic remodeling, barrier vulnerability, and mucosal immune imbalance. The next key question is how these abnormal peripheral inputs reach and affect the central nervous system through interorgan pathways, ultimately promoting the occurrence and progression of remote brain dysfunction. Existing evidence suggests that this process is unlikely to depend on a single pathway, but is more likely to involve the synergistic actions of immune-inflammatory, vagal, and neuroendocrine pathways (Besecker et al., 2020; Kigerl et al., 2020, 2016; Prüss et al., 2017).

It should be emphasized that these three pathways are not mutually independent or unrelated parallel mechanisms (Table 1). More accurately, they should be understood as a pathological network composed of one main axis and two complementary branches. Among them, the immune-inflammatory pathway is the main route by which gut-derived pathological signals affect the brain, primarily converting local intestinal instability into a systemic pro-inflammatory environment capable of affecting remote organs. The vagal pathway is more like a rapid neural relay branch, converting local intestinal abnormalities into neural inputs that are afferent and processed by the central nervous system. The neuroendocrine pathway serves as a background regulatory branch, reinforcing the stability of the first two pathways and their pathological effects under conditions of persistent stress (Besecker et al., 2020; Kigerl et al., 2020, 2016; Prüss et al., 2017).

**Table 1:** Evidence stratification of the proposed gut–brain pathways linking gut-derived pathological signals to remote brain dysfunction after SCI.

Pathway	Direct SCI-related evidence	Cross-disease or mechanistic evidence	Proposed interpretation in this review
Immune-inflammatory pathway	SCI has been associated with gut dysbiosis, intestinal barrier disruption, bacterial translocation, endotoxemia, systemic inflammatory activation, remote brain inflammation, and cognitive or affective alterations.	Studies from systemic inflammation, BBB dysfunction, bacterial vesicles, and gut–brain axis models support the possibility that peripheral inflammatory signals may impair BBB integrity and activate microglia or reactive astrocytes.	This pathway is considered the main axis linking SCI-associated intestinal abnormalities to remote brain dysfunction. However, the complete causal chain from gut barrier disruption to systemic inflammation, BBB impairment, brain neuroinflammation, and behavioral dysfunction still requires stepwise validation in SCI models.
Vagal pathway	Direct SCI evidence mainly shows that high-level SCI can alter gastrointestinal vagal afferent responsiveness, including impaired responses to gut-derived signals such as CCK.	Evidence from colitis, stress-related, depression-related, and ischemic models suggests that vagal afferents can encode inflammatory or microbial signals and relay them to brainstem–limbic networks.	This pathway is proposed as a neural relay branch. Current evidence supports functional remodeling of vagal afferent signaling after SCI, but downstream links from vagal relay signaling to hippocampal/mPFC dysfunction are mainly based on cross-disease gut–brain axis evidence.
Neuroendocrine pathway	SCI can induce abnormal sympathetic–neuroendocrine–adrenal reflexes, dysregulated glucocorticoid and catecholamine exposure, SCI-induced immunodeficiency syndrome, and HPA-axis-related dysfunction.	Evidence from intestinal barrier, chronic stress, and neuroimmune studies suggests that stress hormones may regulate gut barrier resilience, mucosal immunity, hippocampal vulnerability, and mPFC plasticity.	This pathway is proposed as a modulatory amplification branch. Direct SCI evidence supports persistent stress-axis and sympatho-adrenal dysregulation, whereas the complete gut–neuroendocrine–brain chain remains partly inferred from chronic stress and related disease models.

BBB, blood–brain barrier; CCK, cholecystokinin; HPA, hypothalamic–pituitary–adrenal; mPFC, medial prefrontal cortex; SCI, spinal cord injury.

Therefore, this section does not regard these pathways as mutually separate and isolated mechanisms, but conceptualizes them as an interorgan bridging network that is temporally intertwined and ultimately converges on common central consequences. Although the initiation mechanisms of these pathways differ, they all point to a common downstream brain effector stage characterized by amplification of neuroinflammatory networks, impaired synaptic plasticity, and dysfunction of key brain regions. Based on this framework, the following sections first discuss how the immune-inflammatory pathway constitutes the main mechanism by which gut-derived pathological signals enter and affect the brain, then examine how the vagal pathway mediates rapid neural transmission of intestinal abnormalities, and finally analyze how the neuroendocrine pathway maintains and amplifies these pathological processes in the context of persistent stress.

### 3.1 Immune-inflammatory pathway: interorgan amplification of gut-derived inflammatory signals and their central effects

Among the three bridging pathways, the immune-inflammatory pathway currently has the most concentrated evidence and also provides the most readily integrated mechanistic explanation. It should be noted that in direct SCI models, the complete causal chain from intestinal barrier disruption, bacterial translocation, and systemic inflammatory amplification to innate immune activation in the brain and remote brain dysfunction has not yet been validated step by step. Nevertheless, existing studies have provided relatively reliable support from both the gut and the central nervous system. On the one hand, SCI can induce gut microbial dysbiosis, barrier disruption, endotoxemia, and a chronic inflammatory background; on the other hand, SCI itself is associated with inflammatory responses in remote brain regions as well as cognitive and affective alterations (Faden et al., 2016; Wu et al., 2014; Ning et al., 2026; Diaz et al., 2021; Kigerl et al., 2016; O'Connor et al., 2018; Myers et al., 2019). Therefore, based on the current evidence landscape, systemic inflammatory amplification is more appropriately understood as the main mechanism connecting intestinal abnormalities with central effects, rather than as a fully closed causal chain that has already been proven step by step.

Specifically, Section 2 discussed how disruption of intestinal homeostasis after SCI gradually leads to barrier vulnerability, mucosal immune imbalance, and abnormal exposure to microbe-associated molecular patterns (Diaz et al., 2021; Kigerl et al., 2016; O'Connor et al., 2018; Myers et al., 2019). The pathological significance of these changes extends beyond local intestinal inflammation itself. More importantly, they create conditions that allow gut-derived inflammatory signals to continuously enter the circulation. Previous studies have shown that increased intestinal permeability can promote the entry of lipopolysaccharide (LPS) and other microbe-associated molecular patterns into the bloodstream, thereby amplifying systemic inflammatory responses and forming a persistent peripheral pro-inflammatory environment (Kalyan et al., 2022; Kearns, 2024; Zhou et al., 2025). In the context of SCI, this means that the gut is no longer merely an injured organ, but may gradually evolve into a peripheral inflammatory source capable of continuously releasing pro-inflammatory mediators and other inflammatory loads into the circulation. It is at this level that local abnormalities originally confined to the intestinal mucosal interface may be further amplified into a systemic inflammatory background sufficient to affect the brain through interorgan routes.

Once this systemic pro-inflammatory environment is formed, whether gut-derived inflammatory signals can continuously act on the brain depends to a large extent on changes in the permeability of the peripheral–central interface. The blood–brain barrier (BBB) is the most important relay structure in this process. Existing studies show that pro-inflammatory cytokines such as TNF-α and IL-6 can directly act on brain microvascular endothelial cells, inhibit the expression of tight junction proteins such as occludin and claudin-5, and disrupt BBB integrity (Rochfort et al., 2015; Aslam et al., 2012). In the logic of this section, BBB vulnerability should not be regarded merely as a concomitant injury phenomenon, but should be viewed as a key relay step by which gut-derived pathological signals enter the common downstream brain effector stage from the peripheral inflammatory environment. In other words, once BBB function is impaired, the ability of the brain parenchyma to defend against abnormal peripheral signals declines, making pro-inflammatory signals that originally had difficulty entering the central nervous system more likely to penetrate and be amplified.

In addition to soluble pro-inflammatory mediators, vesicle-mediated cargo delivery mechanisms may further increase the efficiency with which gut-derived pathological signals cross the peripheral–central interface. In recent years, bacterial extracellular vesicles (bEVs) and outer membrane vesicles (OMVs) have increasingly been regarded as important carriers of long-distance cross-boundary communication along the gut–brain axis (Pirolli et al., 2021). Recent evidence shows that gut-derived bacterial vesicles can carry LPS across the BBB and trigger inflammation-related signaling programs after being taken up by microglia in the brain (Zhao et al., 2025). It should be emphasized that, in the context of SCI, this does not yet mean that vesicle-mediated delivery has been established as an independent and complete pathogenic chain. However, this mechanism at least suggests that the entry of gut-derived pathological signals into the brain does not depend entirely on diffusion of free inflammatory mediators. Vesicle-mediated delivery of pro-inflammatory cargo may also constitute an important supplementary form of humoral input. Therefore, soluble pro-inflammatory factors and vesicle-mediated cargo delivery are more likely to act in parallel rather than replace each other, jointly promoting the continuous input of gut-derived inflammatory signals into the brain immune microenvironment.

After abnormal peripheral signals successfully act on the central microenvironment, microglia may be among the earliest and most critical innate immune response cells. It should be noted that microglial activation after SCI may arise from at least two partially overlapping sources. One is directly related to central injury-associated inflammatory signaling after SCI, whereas the other may reflect modulation by peripheral immune activation, barrier disruption, microbial products, and altered metabolites. The latter is particularly relevant to the gut–brain axis framework proposed in this review, because it emphasizes how gut-derived pathological signals may reshape the inflammatory state of remote brain regions rather than simply reflecting local spinal cord injury-related inflammation.

Under physiological conditions, microglia maintain a highly ramified morphology and continuously monitor the brain parenchyma to maintain homeostasis. However, in the systemic inflammatory environment secondary to SCI, this homeostatic program may gradually be disrupted (Faden et al., 2016). Existing evidence indicates that gut dysbiosis can activate microglia and promote abnormal synaptic pruning in the hippocampal CA1 region, which is closely related to cognitive decline (Krishnapriya et al., 2025). Further evidence shows that, in the context of SCI, gut microbiota disturbance may be accompanied by activation of the TLR4/MyD88 pathway (Rong et al., 2021). Combined with findings from cross-disease studies that gut-derived inflammation and brain innate immune responses may share TLR4/NF-κB-like inflammatory signaling programs (Zhou et al., 2025), these observations suggest that peripheral inflammation and brain immune activation may involve partially overlapping inflammatory transduction frameworks. Therefore, when gut-derived pathological signals persist after SCI, microglia may respond to abnormal peripheral inflammatory inputs through pattern-recognition and inflammatory transcription pathways such as TLR4/NF-κB, thereby amplifying these signals and promoting the formation of a pro-inflammatory brain microenvironment. In combination with recent authoritative reviews, microglia are very likely to be a key mediating node that converts gut-derived inflammatory input into responses at the brain effector stage (Keane et al., 2025; Krishnapriya et al., 2025).

The pathological significance of the immune-inflammatory pathway is not limited to isolated microglial activation, but may further drive the cascade amplification of multicellular inflammatory networks in the brain. Existing studies show that sustained pro-inflammatory activation of microglia can induce the formation of A1-like reactive astrocytes through key signals such as IL-1α, TNF-α, and C1q (Liddelow et al., 2017). These reactive astrocytes may further amplify the neurotoxic environment, aggravate the spread of inflammation, and weaken the capacity to maintain brain homeostasis (Li et al., 2026; Radford-Smith et al., 2025). Therefore, current evidence suggests that the immune-inflammatory pathway may establish a brain inflammatory effector stage centered on persistent microglial activation and the emergence of reactive astrocytes, thereby providing an important upstream driver for subsequent abnormal synaptic pruning and injury to cognitive–affective networks. Importantly, intestinal abnormalities after SCI are not a one-time insult, but may constitute a long-lasting peripheral pathological source. This persistent input itself may be an important factor that prevents spontaneous resolution of pro-inflammatory brain networks and ultimately promotes the development of chronic remote brain dysfunction.

In summary, the core role of the immune-inflammatory pathway lies in completing the interorgan bridge from local intestinal abnormalities to systemic inflammatory amplification, then to BBB impairment, and ultimately to innate immune activation in the brain. Although this complete chain has not yet been validated step by step in direct SCI models, existing evidence from individual SCI studies, together with highly consistent mechanistic support from related disease models, is sufficient to support its status as the main pathway in this section. Meanwhile, gut-derived pathological signals do not enter the brain only through humoral inflammatory delivery. In addition to the immune-inflammatory main axis, local intestinal abnormalities after SCI may also be rapidly encoded as neural signals through vagal afferent pathways and further affect brainstem–limbic regulatory networks. Therefore, besides humoral inflammatory delivery, another important route by which gut-derived pathological signals reach the brain may be rapid neural transmission via the vagal pathway.

**Figure 2:**
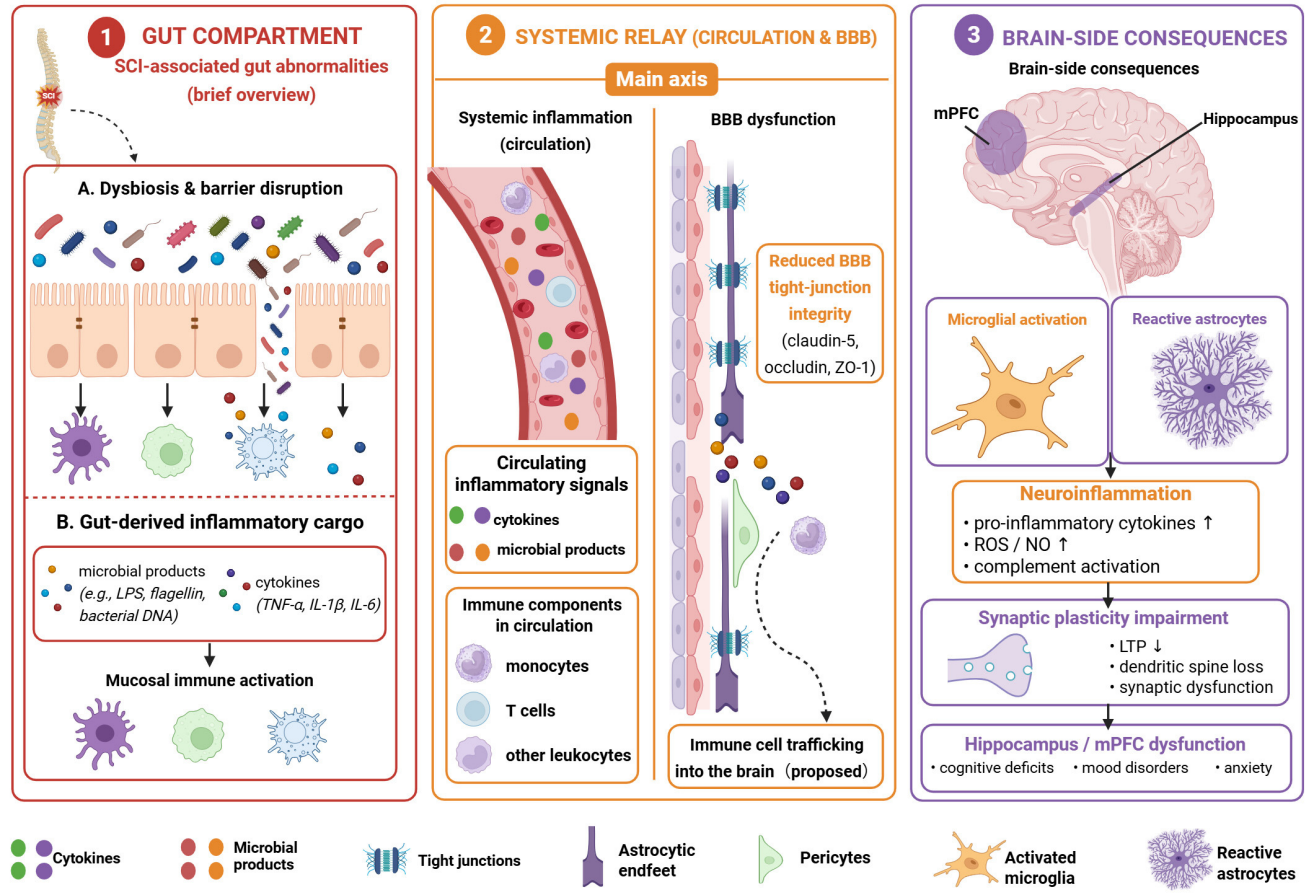
Immune-inflammatory main axis linking gut-derived pathological signals to remote brain dysfunction after SCI. SCI-associated gut abnormalities, including dysbiosis, epithelial barrier disruption, mucosal immune activation, and the release of microbial products and cytokines, may promote systemic inflammatory amplification. Circulating inflammatory signals can impair BBB integrity and facilitate immune-related communication with the brain. This process may activate microglia and reactive astrocytes, leading to neuroinflammation, synaptic plasticity impairment, and dysfunction of the hippocampus and mPFC. BBB, blood–brain barrier; mPFC, medial prefrontal cortex; ROS, reactive oxygen species; NO, nitric oxide; LTP, long-term potentiation.

The proposed immune-inflammatory main axis linking gut-derived pathological signals to remote brain dysfunction after SCI is illustrated in Figure 2.

### 3.2 Vagal pathway: a neural bridge transmitting abnormal intestinal signals to the central nervous system

Compared with the immune-inflammatory pathway, which mainly depends on humoral inflammatory delivery, the vagal pathway represents a more rapid neural relay mechanism. Its significance lies not in replacing the immune-inflammatory main axis, but in emphasizing that local intestinal abnormalities after SCI do not necessarily need to be completely converted into circulating inflammatory factors before they can affect the central nervous system. Instead, some abnormal signals may be encoded as neural signals through vagal afferent pathways and transmitted to the brainstem and its ascending regulatory networks. Therefore, the vagal pathway can be understood as a neural relay branch connecting the intestinal sensory interface with central integrative systems. It complements the immune-inflammatory main axis and jointly participates in the interorgan transmission of gut-derived pathological signals to the brain.

Under physiological conditions, the vagus nerve is one of the most important bidirectional communication pathways in the gut–brain axis, and its afferent fibers play a key role in transmitting the local intestinal state to the central nervous system in real time. The cell bodies of vagal afferent neurons are mainly located in the nodose ganglion, and their peripheral terminals are distributed across different layers of the gastrointestinal tract, where they can sense multiple inputs such as mechanical distension, nutritional stimuli, gut hormones, and immune-related signals (Latorre et al., 2016; Yu et al., 2020). Recent studies further suggest that enteroendocrine cells (EECs) are key components of the intestinal sensory interface. These cells can integrate information from luminal nutrients, microbial signals, and the local inflammatory environment, and act on vagal terminals by releasing signaling molecules such as cholecystokinin (CCK), glucagon-like peptide-1 (GLP-1), peptide YY (PYY), and 5-hydroxytryptamine (5-HT), thereby converting local intestinal changes into neural inputs that can be transmitted to and processed by the central nervous system (Latorre et al., 2016; Yu et al., 2020; Ohara and Hsiao, 2025). Therefore, the vagus nerve does not merely mediate gastrointestinal reflexes or satiety signals, but serves as an integrative sensory bridge capable of processing mechanical, chemical, endocrine, and immune information.

In the context of SCI, this vagal sensory system, which is usually used for homeostatic monitoring, may undergo remodeling. As described in Section 2, high-level SCI can induce dysfunction of gastric vagal afferents, suggesting that the gut may gradually shift from a homeostatic sensory interface to an abnormal signal-generating interface. This provides a direct basis for discussing the vagal pathway in this chapter. Existing SCI studies show that after injury, the response of gastric vagal afferents to CCK stimulation is significantly reduced, indicating that although SCI does not directly transect the vagus nerve itself, it may indirectly induce neuropathy-like changes in gastric vagal afferents by altering the local gastrointestinal environment and brainstem reflex integration (Besecker et al., 2020; Blanke et al., 2021). Therefore, in the pathological cascade of SCI, the key question regarding the vagal pathway is not whether the vagus nerve is physically interrupted, but whether the information it senses and transmits has become abnormal. The source of this abnormal sensory input is precisely the intestinal background established in Section 2: intestinal dysmotility, gut dysbiosis, depletion of SCFAs, barrier disruption, and persistent local inflammation jointly alter the physicochemical and immune microenvironment of the intestinal lumen and mucosa. In other words, after SCI, the gut does not merely develop local inflammation or microbial dysbiosis; its local sensory interface may have undergone overall remodeling.

On this basis, aberrant local inflammatory signals do not necessarily need to first enter the circulation in humoral form in order to affect the central nervous system. Some signals may be encoded at the level of vagal afferents. Recent studies have shown that vagal sensory neurons are not merely passive detectors of the inflammatory environment, but can generate differentiated real-time neural activity responses to specific pro-inflammatory cytokines. Huerta et al. found that vagal sensory neurons in the nodose ganglion exhibit selective and differentiated response patterns to inflammatory cytokines such as TNF-α and IL-1β, and that these encoding features are further remodeled under colitis conditions (Huerta et al., 2025). The significance of this finding is that inflammatory information may be precisely encoded at the vagal afferent level, rather than only being passively transmitted after entering the circulation in humoral form. Therefore, when persistent local intestinal inflammation and immune abnormalities exist after SCI, the vagus nerve is unlikely to transmit only non-specific visceral discomfort signals, but may convert the abnormal immune state into neural inputs that can be processed by central circuits with higher specificity.

At the functional level, increasing evidence also suggests that vagal integrity is an important condition for microbiota-related abnormalities to affect brain function. In stress-related models, Siopi et al. (2023) found that depression-like behaviors and hippocampal abnormalities induced by microbiota alterations require an intact vagus nerve to be fully expressed; after vagotomy, the effects of microbiota alterations on behavioral phenotypes and neurogenesis were significantly attenuated. Although this finding does not come from an SCI model, its value lies in showing that the vagus nerve is not merely a background factor passively involved in gut–brain communication, but may be a key pathway through which microbiota-related abnormal signals are transmitted to brain networks involved in behavioral regulation. Another study showed that extracellular vesicles from the depression-associated strain *Escherichia fergusonii* can induce vagus nerve-mediated neuroinflammation, while vagotomy can significantly reduce their transport to the hippocampus (Ma et al., 2024). In addition, in a cerebral ischemia model, the microbiota-derived metabolite H_2_S was shown to participate in the regulation of microglial polarization through vagus nerve–TRPV1 signaling (Ni et al., 2022). These findings suggest that the vagal pathway should not be regarded merely as an upstream sensory branch; instead, it may help determine whether gut-derived pathological signals can effectively affect brain regulatory networks and be further translated into inflammatory and behavioral consequences.

On the central side, abnormal vagal inputs first project to the nucleus tractus solitarius (NTS) in the brainstem, and subsequently further affect the hypothalamus, autonomic brainstem nuclei, and limbic system networks. Existing reviews indicate that vagal sensory input is involved not only in gastrointestinal reflexes and metabolic homeostasis, but also in transmitting signals from the NTS to higher brain regions, thereby affecting stress responses, emotional processing, and memory-related functions (Yu et al., 2020; Kuijer and Steenbergen, 2023). Therefore, in the context of remodeling of the intestinal sensory interface after SCI, the pathological significance of the vagal pathway should not be understood merely as abnormal regulation of gastrointestinal reflexes. More importantly, it may continuously convert local intestinal abnormalities into neural inputs that can be processed by central circuits, thereby altering the regulatory background of the brainstem–hypothalamic–limbic system and increasing the responsiveness of brain tissue to stress and peripheral inflammatory signals. Compared with the immune-inflammatory pathway, the vagal pathway emphasizes neural and temporally faster signal transmission; together, the two determine the manner and temporal pattern by which gut-derived pathological signals reach the brain.

Compared with the immune-inflammatory pathway, direct evidence for a complete causal chain between abnormal vagal afferent signaling and remote brain inflammation, cognitive impairment, or affective disorders in SCI models remains relatively limited. Existing SCI studies have mainly demonstrated that high-level SCI alters the responsiveness and physiological properties of gastrointestinal vagal afferents (Besecker et al., 2020; Blanke et al., 2021). However, how these abnormal signals further affect the hippocampus, medial prefrontal cortex, or cognitive–affective networks still relies mainly on mechanistic evidence from the broader gut–brain axis field and other disease models (Huerta et al., 2025; Siopi et al., 2023; Ma et al., 2024; Ni et al., 2022; Kuijer and Steenbergen, 2023). Therefore, a more cautious conclusion is that the vagal pathway may serve as a bridging pathway that rapidly encodes and transmits neural signals of local intestinal abnormalities after SCI. Although it may not independently determine the full spectrum of brain injury outcomes, it may significantly alter the central regulatory background and provide an important neural basis for the amplification of the immune-inflammatory axis in the brain and the subsequent occurrence of brain-region dysfunction. Based on this view, the next section will further discuss how the neuroendocrine pathway, as a persistent stress-related regulatory background, maintains and enhances the pathological effects of the first two pathways.

**Figure 3:**
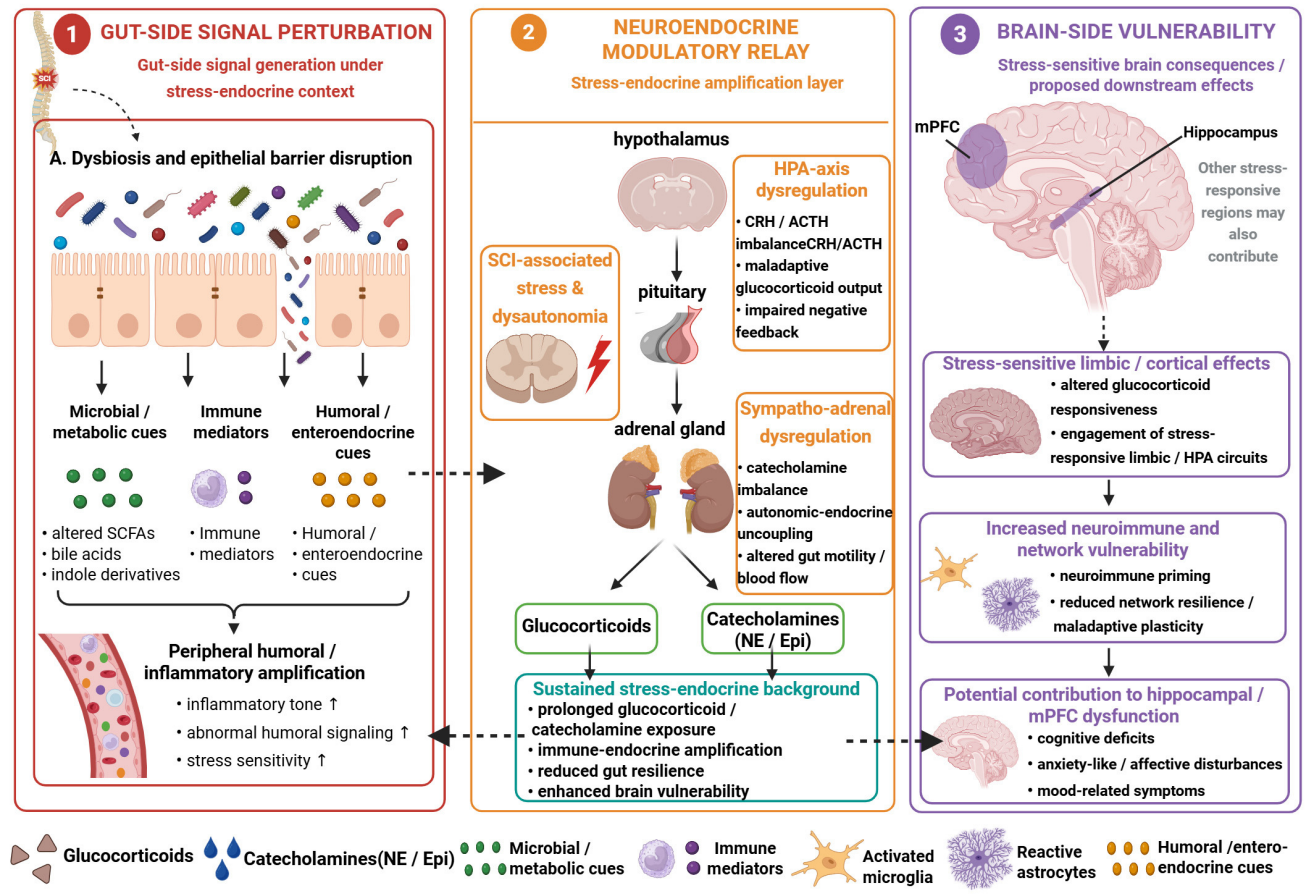
Proposed vagal pathway linking gut-derived pathological signals to remote brain dysfunction after SCI. After SCI, gut dysbiosis, barrier disruption, microbial products, altered metabolites, and enteroendocrine cell-derived signals may alter vagal afferent signaling. These local intestinal abnormalities may be relayed through the nodose ganglion and nucleus tractus solitarius to brainstem, hypothalamic, and limbic circuits. The vagal pathway may therefore serve as a neural relay branch that modulates central neuroimmune responses, synaptic plasticity, and hippocampal/mPFC-related dysfunction. This figure should be interpreted as a proposed pathway: direct SCI-specific evidence currently supports functional remodeling of gastrointestinal vagal afferent responsiveness, whereas several downstream links from vagal relay signaling to hippocampal/mPFC dysfunction are based on cross-disease gut–brain axis evidence and require further SCI-specific validation. EEC, enteroendocrine cell; NTS, nucleus tractus solitarius; DMV, dorsal motor nucleus of the vagus; CCK, cholecystokinin; GLP-1, glucagon-like peptide-1; PYY, peptide YY; 5-HT, 5-hydroxytryptamine; mPFC, medial prefrontal cortex.

The proposed vagal relay pathway linking gut-derived pathological signals to remote brain dysfunction after SCI is summarized in Figure 3.

### 3.3 Neuroendocrine pathway: stress-axis dysregulation and gut–brain pathological coupling

If the immune-inflammatory pathway mainly emphasizes the interorgan transmission of gut-derived pathological signals through humoral routes, and the vagal pathway emphasizes the rapid neural transmission of local intestinal abnormalities, then the neuroendocrine pathway reflects the long-term regulatory effect of the persistent stress state after SCI on intestinal homeostasis and brain vulnerability. Recent psychoneuroimmunoendocrine studies of SCI further suggest that neuroendocrine dysregulation should not be regarded as a mere accompanying phenomenon, but rather as an important component of the chronic systemic consequences after injury (Ortega et al., 2023). The key role of this pathway is not to directly mediate all abnormal signal transmission, but to provide a persistent stress-related regulatory background for the pathological processes described above. The delayed and persistent amplification of remote brain dysfunction after SCI may not depend entirely on a one-time inflammatory input, but is also closely related to persistent neuroendocrine dysregulation after injury. Therefore, the neuroendocrine pathway can be regarded as a regulatory branch that maintains and amplifies abnormal states. By simultaneously affecting the ability of the intestine to restore homeostasis and the stress vulnerability of the brain, it may participate in the long-term amplification of the pathological effects of the first two pathways.

In direct SCI models, the most important evidence supporting this pathway comes from the study by Prüss et al. This study showed that SCI can induce an abnormal sympathetic–neuroendocrine–adrenal reflex, leading to dysregulated exposure patterns of glucocorticoids and catecholamines and further remodeling peripheral immune homeostasis (Prüss et al., 2017). More importantly, this work not only revealed the neuroendocrine basis of systemic immunosuppression after SCI, but also proposed the conceptual framework of SCI-induced immunodeficiency syndrome (SCI-IDS), providing a key starting point for understanding gut–brain pathological coupling after high-level SCI (Prüss et al., 2017). A recent multi-tissue transcriptomic study further suggested that SCI-IDS is not a single immune phenotype, but is accompanied by complex remodeling of neuroendocrine regulatory networks. After high-level SCI, hypothalamus-related inflammatory pathways, adrenal rhythm regulation, and peripheral hormone exposure patterns may all undergo persistent perturbation, providing more direct SCI-related evidence for sustained post-injury stress-axis dysregulation (Zeng et al., 2023). Clinical studies also suggest that some individuals with chronic SCI have hypothalamic–pituitary–adrenal (HPA) axis dysfunction, indicating that this imbalance is not limited to the acute phase, but may extend into the chronic phase (Cuce et al., 2019). These findings indicate that the pathological impact of SCI extends beyond local interruption of neural conduction and may further expand central injury into persistent systemic neuroendocrine disturbance through abnormal stress-axis responses.

On the intestinal side, abnormal exposure to glucocorticoids and catecholamines may not mainly manifest as direct injury. More importantly, it may remodel the regulatory background of the intestinal barrier and mucosal immunity. A classic study showed that glucocorticoids can regulate intestinal epithelial tight junction barrier function and affect TNF-α-induced barrier disruption (Boivin et al., 2007). A recent systematic review further indicated that glucocorticoid signaling is closely related to mitochondrial function, endothelial homeostasis, and intestinal barrier integrity, suggesting that its mode of action is more akin to resetting the regulatory threshold of barrier function rather than simply weakening the barrier in a unidirectional mechanical manner (Meduri and Psarra, 2026). This is particularly important in the SCI context, because it suggests that the role of the neuroendocrine pathway on the intestinal side may not be limited to inducing transient mucosal vulnerability. Instead, by altering epithelial renewal, tight junction regulation, and local immune tolerance, this pathway may make the intestinal dysbiosis, depletion of SCFAs, and barrier vulnerability described in Section 2 more likely to persist. From this perspective, the neuroendocrine pathway and the immune-inflammatory pathway are not mutually independent. The neuroendocrine pathway may act as an upstream factor that continuously reduces the ability of the intestine to restore homeostasis; the immune-inflammatory pathway then converts this instability into systemic inflammatory signals capable of affecting distant organs.

On the brain side, long-term abnormal glucocorticoid exposure and stress-related signals may also constitute important pathological regulatory factors. Existing studies suggest that chronic stress states can not only alter hippocampal inflammatory susceptibility and glial cell states, but also weaken neurotrophic support and neural plasticity, making related brain regions more prone to persistent dysfunction. Chen et al. (2024) showed that chronic stress can alter complement C5aR1 signaling and stress reactivity, accompanied by hippocampal microglia-related changes, thereby suggesting an observable biological link among stress, immunity, and hippocampal vulnerability. Meanwhile, a recent review on the prefrontal cortex systematically summarized dendritic spine remodeling, changes in neural connectivity, and cognitive or affective dysfunction in the medial prefrontal cortex (mPFC) under chronic stress, emphasizing that the prefrontal cortex is a key target region for stress-induced maladaptive neuroplasticity (Ren et al., 2025). Combined with the aforementioned multi-tissue SCI study, current evidence suggests that the hippocampus and medial prefrontal cortex are repeatedly regarded as key vulnerable brain regions after SCI not only because they are sensitive to inflammation, but also because they are highly susceptible to abnormal stress hormone exposure.

Therefore, the role of neuroendocrine abnormalities in remote brain dysfunction after SCI is mainly reflected in the persistent regulation of existing pathological processes. Abnormal exposure to glucocorticoids and catecholamines may act simultaneously on the intestine and the brain. In the intestine, these mediators may reduce the ability of the barrier and mucosal immune system to restore homeostasis. In the brain, they may increase the susceptibility of the hippocampus and medial prefrontal cortex to stress, inflammation, and abnormal neural inputs. Therefore, the neuroendocrine pathway is more likely to promote the stabilization and chronic progression of remote brain dysfunction by maintaining a persistent stress state and amplifying the aforementioned pathological signals.

It should be noted that direct SCI evidence for the complete chain linking neuroendocrine abnormalities, persistent disruption of intestinal homeostasis, and damage to specific brain regions remains relatively limited. Existing studies have confirmed the presence of abnormal neuroendocrine reflexes and HPA-axis-related dysregulation after SCI, whereas evidence for abnormal intestinal barrier regulation and the high sensitivity of the hippocampus and medial prefrontal cortex to stress-related backgrounds mainly comes from chronic stress and related disease models. Nevertheless, these findings still support regarding neuroendocrine abnormalities as an important background amplification factor for remote brain dysfunction after SCI. Although this pathway may not independently determine all brain injury outcomes, it can further stabilize these pathological effects through a persistent stress state and promote their chronic progression.

In summary, these three pathways indicate that gut-derived pathological signals after SCI do not affect remote brain function through a single mechanism. The immune-inflammatory pathway, as the main axis, converts intestinal instability into a systemic inflammatory background and transmits it to the brain. The vagal pathway, as a neural relay branch, rapidly encodes local intestinal abnormalities and transmits them to the brainstem–limbic network. The neuroendocrine pathway, as a regulatory branch, reduces the ability of the intestine to restore homeostasis and increases brain vulnerability under persistent stress. Although the initiating mechanisms of these pathways differ, they ultimately tend to converge in the brain at a common downstream brain effector stage, characterized by activation of neuroinflammatory networks, impaired synaptic plasticity, and brain-region dysfunction. Among them, the hippocampus and medial prefrontal cortex may be particularly vulnerable nodes because of their dual sensitivity to stress hormones and inflammatory signals. This multi-pathway coupling framework not only helps explain why remote brain dysfunction after SCI shows delayed onset and persistent amplification, but also provides a theoretical basis for multi-target intervention strategies based on the gut–brain axis, which will be discussed in the next chapter.

**Figure 4:**
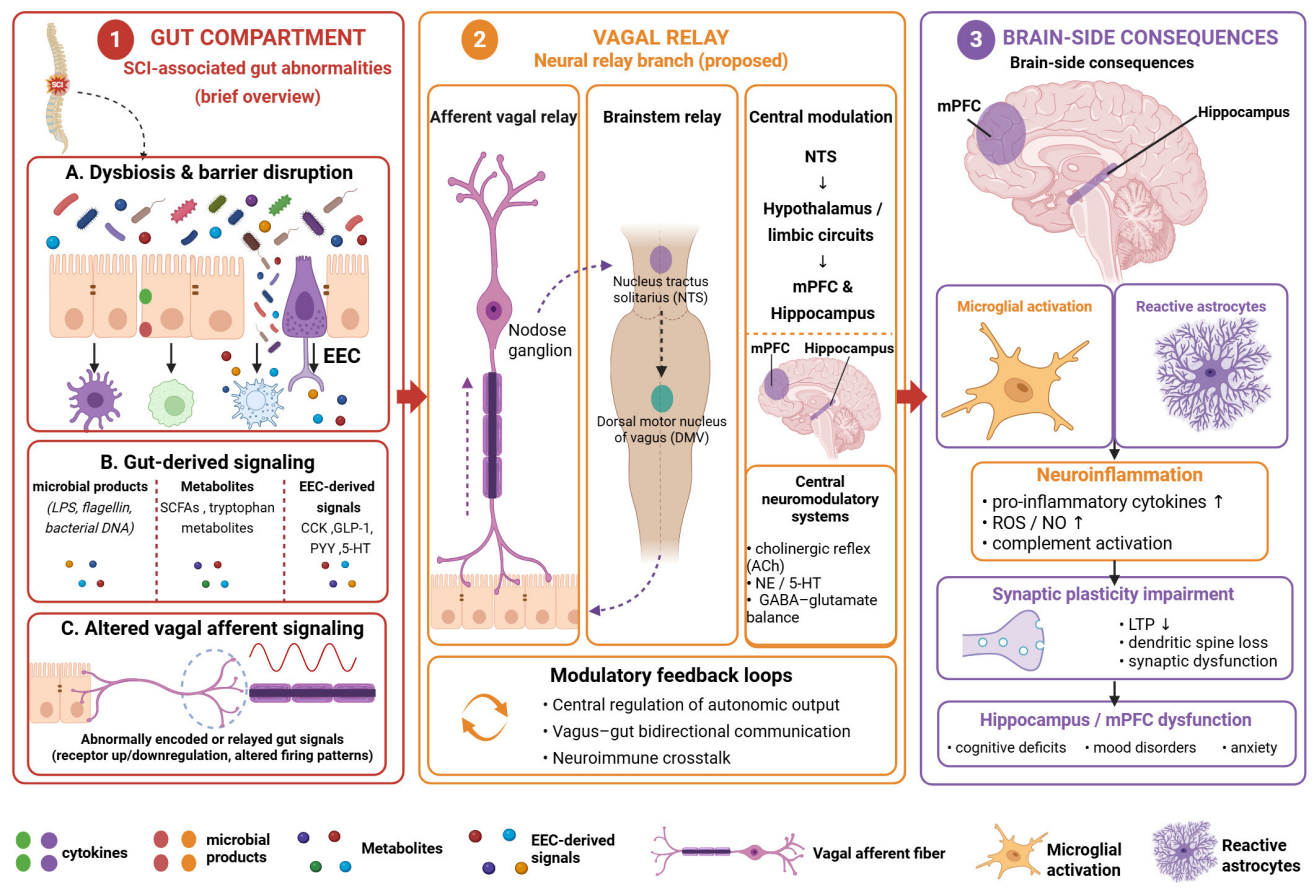
Proposed neuroendocrine modulatory relay linking gut-derived pathological signals to brain-side vulnerability after SCI. SCI-associated stress and dysautonomia may induce HPA-axis and sympatho-adrenal dysregulation, resulting in abnormal glucocorticoid and catecholamine exposure. These neuroendocrine changes may amplify peripheral humoral and inflammatory signaling, reduce gut resilience, and increase stress sensitivity in vulnerable brain regions such as the hippocampus and mPFC. This pathway is proposed as a modulatory amplification layer that enhances neuroimmune priming, maladaptive plasticity, and cognitive or affective dysfunction after SCI. Direct SCI evidence supports persistent stress-axis and sympatho-adrenal dysregulation after injury; however, the complete gut–neuroendocrine–brain chain linking intestinal barrier regulation, stress hormone exposure, and hippocampal/mPFC dysfunction remains partly inferred from chronic stress and related disease models and requires further SCI-specific validation. HPA, hypothalamic–pituitary–adrenal; CRH, corticotropin-releasing hormone; ACTH, adrenocorticotropic hormone; NE, norepinephrine; Epi, epinephrine; mPFC, medial prefrontal cortex.

The neuroendocrine modulatory relay and its contribution to remote brain vulnerability after SCI are summarized in Figure 4.

## 4 Intervention strategies and translational prospects based on the gut–brain axis pathological cascade

As described above, remote brain dysfunction after SCI is not driven by a single pathological event, but is the result of sequential interactions among disruption of intestinal homeostasis, abnormalities in interorgan relay pathways, and dysregulation at the common downstream brain effector stage. Section 2 shows that SCI can gradually generate persistent gut-derived pathological signals through autonomic and neuroendocrine imbalance, intestinal motility disorders, microbial remodeling, and barrier vulnerability. Section 3 further discussed how these signals act on remote brain regions through immune-inflammatory, vagal, and neuroendocrine pathways, ultimately leading to amplification of neuroinflammation, impaired synaptic plasticity, and cognitive or affective dysfunction. Based on this pathological cascade, intervention strategies should not be limited to a single target, but should be developed around three levels: the generation of source pathological signals, their transmission through interorgan relay pathways, and their amplification at the common downstream brain effector stage.

At present, the level of evidence for interventions targeting the gut–brain axis remains uneven. Overall, upstream source-control strategies, especially those aimed at microbiota remodeling, metabolite restoration, and intestinal barrier repair, have obtained some direct support in SCI animal models. In contrast, direct regulation of relay pathways, particularly interventions targeting the vagal interface and the neuroendocrine stress background, is still mainly based on mechanistic inference or evidence from other disease models in the SCI context. Protection of the common downstream brain effector stage is mainly a complementary strategy aimed at limiting terminal pathological amplification. Therefore, this section does not attempt to list all potential therapies in parallel, but instead follows the pathological cascade described above to summarize the most representative intervention targets and their translational significance.

From a translational perspective, the difficulty in moving these strategies toward clinical application is not merely the lack of potential targets. More importantly, several common bottlenecks remain in the evidence chain. Existing studies often focus on local intestinal inflammation, microbial structure, or recovery of motor function, while validation of core outcomes such as remote brain inflammation and cognitive and affective phenotypes remains insufficient. The dominant pathological processes and optimal intervention windows at different disease stages remain unclear, making it difficult to determine how upstream control, relay pathway blockade, and terminal protection should be temporally coordinated. In addition, most existing evidence still comes from animal models or cross-disease mechanistic support, and SCI-specific direct intervention studies that can validate the complete chain from intestinal abnormalities to interorgan transmission and then to brain-region injury remain limited. Therefore, the intervention strategies discussed in this section should be understood as a stratified translational framework based on current mechanistic evidence, rather than mature and established therapeutic pathways.

### 4.1 Upstream source control: restoring intestinal homeostasis and reducing abnormal signal release

Based on the pathological framework described above, the intestine after SCI is not merely a secondarily affected organ, but an upstream source of abnormal signals in the pathological cascade of remote brain dysfunction. Therefore, from an intervention perspective, the focus may not necessarily be direct targeting of terminal brain pathology, but rather reducing the formation and release of gut-derived pathological signals. The core goal of upstream source control is not only to improve local gastrointestinal symptoms, but also to restore intestinal homeostasis, thereby reducing the pathological inputs continuously received by the immune-inflammatory axis, vagal neural relay, and neuroendocrine regulatory branch (Kigerl et al., 2020, 2016; Diaz et al., 2021).

At this level, microbiota remodeling and metabolite restoration are two important directions. Existing preclinical studies suggest that fecal microbiota transplantation (FMT) can to some extent improve the decrease in microbial diversity and compositional imbalance after SCI, accompanied by reductions in local intestinal inflammation and systemic inflammatory levels (Xi et al., 2024). Some cross-disease studies also suggest that microbiota reconstruction may be associated with alleviation of brain inflammation and improvement in cognitive or behavioral outcomes. However, in the SCI context, such downstream brain effects still lack sufficient direct validation (Dong et al., 2024). Compared with FMT, probiotic intervention places greater emphasis on improving the intestinal microbial environment and mucosal barrier status in a relatively controllable manner. Given the presence of gut dysbiosis and disruption of intestinal homeostasis after SCI, such strategies have theoretical rationality (Kigerl et al., 2016; Pagan-Rivera et al., 2024).

In addition to regulating microbial composition, restoring short-chain fatty acids (SCFAs), especially butyrate, is also of great significance. SCFAs not only participate in maintaining mucosal immune tolerance, but can also promote intestinal epithelial barrier repair and stabilize tight junction-related molecules (Smith et al., 2013; Wang et al., 2012; Xu et al., 2023; Jing et al., 2023). In SCI models, butyrate-related interventions have also shown certain anti-inflammatory and neuroprotective potential (Ganggang et al., 2025). Therefore, upstream source control ultimately depends on restoring intestinal barrier function and mucosal immune homeostasis. Its true value does not lie in improving a single local indicator, but in reducing subsequent interorgan pathological inputs at the source (Kigerl et al., 2016; Smith et al., 2013; Wang et al., 2012; Myers et al., 2019).

However, relying solely on upstream source control may be insufficient to completely prevent the occurrence and progression of remote brain dysfunction after SCI. Once systemic inflammation, abnormal neural relay signals, and a persistent stress state have already formed, the effects of restoring intestinal homeostasis may lag behind. Therefore, while reducing the release of gut-derived pathological signals, it is also necessary to consider whether key regulatory nodes exist within the interorgan relay process itself. It is worth noting that although many current upstream interventions can improve gut dysbiosis, barrier injury, or systemic inflammation, whether they can simultaneously improve remote brain inflammation and cognitive or affective outcomes still lacks sufficient support from direct SCI evidence.

### 4.2 Regulation of interorgan relay pathways: blocking the transmission of gut-derived pathological signals to the brain

Compared with upstream source control, interventions directly targeting interorgan relay pathways remain relatively limited in SCI models, and a large portion of the existing evidence comes from mechanistic studies in other neurological or inflammatory disease models. Therefore, the strategies discussed in this section should be regarded as potential intervention directions derived from the pathological cascade, rather than mature methods that have been fully validated in SCI. From this perspective, the goal of interventions at the relay level is to limit continued transmission and amplification of pathological signals after they have already been generated.

At the level of the immune-inflammatory axis, the focus of relay regulation is not mainly to reduce the formation of intestinal abnormal signals, but to block inflammatory mediators that have entered the circulation from crossing the blood–brain barrier (BBB) and entering the brain parenchyma. Based on this logic, reducing the peripheral pro-inflammatory burden, alleviating systemic inflammation, and protecting BBB integrity can be regarded as important directions. Existing studies suggest that pro-inflammatory mediators such as TNF-α and IL-6 can not only contribute to a persistent peripheral pro-inflammatory environment (Kalyan et al., 2022; Kearns, 2024; Zhou et al., 2025), but may also act on brain microvascular endothelial cells and cause changes in BBB permeability (Rochfort et al., 2015; Aslam et al., 2012), thereby creating conditions for abnormal inflammatory signals to enter the brain. Therefore, drugs with anti-inflammatory and potential barrier-protective effects, such as minocycline, may to some extent help limit this abnormal transmission (Hu et al., 2025). However, in the SCI context, such strategies mainly represent interception of the relay node involving peripheral inflammation, BBB vulnerability, and central delivery. Their optimal therapeutic window and long-term safety still need to be further clarified.

At the level of the vagal neural relay branch, the key intervention target is modulation of this vagal sensory interface. Based on the pathological logic described in Section 3, vagus nerve stimulation (VNS) and other autonomic neuromodulation methods may indirectly weaken the neural encoding and transmission of gut-derived pathological signals by regulating the cholinergic anti-inflammatory reflex and the peripheral inflammatory background (Tracey, 2002; Pavlov and Tracey, 2019; Xie et al., 2024). However, current evidence in the SCI context remains limited. Therefore, VNS should be more cautiously described as a theoretically promising strategy for regulating the relay interface, rather than a treatment method that has been fully validated and established.

At the level of the neuroendocrine regulatory branch, relay-level intervention mainly involves management of the persistent stress state, rather than direct blockade of a single pathological signal. As described in Section 3, abnormal sympathetic–neuroendocrine–adrenal reflexes and HPA-axis-related network remodeling after SCI may continuously amplify immune-inflammatory inputs and abnormal neural relay signals by reducing the ability of the intestine to restore homeostasis and increasing the vulnerability of the brain to stress. Therefore, regulating HPA-axis imbalance, abnormal glucocorticoid exposure, and persistent stress-related states can be regarded as potential auxiliary intervention directions (Prüss et al., 2017; Zeng et al., 2023; Cuce et al., 2019; Meduri and Psarra, 2026). However, in the SCI context, direct interventional evidence for this pathway is particularly limited and currently remains mainly based on mechanistic plausibility and inference from the pathological framework. Therefore, it is more appropriate to regard this pathway as a background regulatory layer in relay-level modulation, rather than the most mature current intervention target.

Overall, regulation of interorgan relay pathways is not a replacement for upstream source control, but an important complement to it. Especially when systemic inflammation, abnormal neural relay signals, and persistent stress have already formed after SCI, restoring intestinal homeostasis alone may not reverse all interorgan pathological processes in a timely manner. Therefore, constructing relay-level interventions around the immune-inflammatory axis, the vagal interface, and the neuroendocrine stress background has clear pathological and translational logic.

### 4.3 Terminal protection and prospects for combined intervention

Although upstream source control and relay pathway regulation can respectively reduce the abnormal signal burden at the intestinal source and during interorgan transmission, once the pathological process has converged at the common downstream brain effector stage, they may still be insufficient to completely reverse remote brain dysfunction. As described in Section 3, gut-derived pathological signals after SCI do not remain confined to the periphery, but further converge in the brain at the common downstream brain effector stage, whose core features include persistent microglial activation, reactive astrocyte formation, impaired synaptic plasticity, and dysfunction of the hippocampus and medial prefrontal cortex. Therefore, the significance of terminal protection is not to replace the first two intervention levels, but to limit and buffer final functional injury after pathological amplification has already been initiated in the brain.

From this perspective, terminal protection first focuses on regulation of neuroglial networks. Existing studies suggest that microglia are not only early amplification nodes of SCI-related neuroinflammation, but may also continue to participate in maintaining a pro-inflammatory brain microenvironment during the chronic phase. Therefore, inhibiting excessive microglial activation or functionally reprogramming microglia has become a potential strategy worthy of attention (Wang et al., 2025; Cavalcanti et al., 2025). At the same time, the goal of terminal protection should not be limited to reducing inflammatory markers, but should further consider synaptic integrity, neural network stability, and preservation of cognitive or affective functions related to key brain regions. Recent studies show that interventions targeting the inflammatory brain environment after SCI can not only alleviate brain inflammation, but may also reverse related cognitive decline. Clinical evidence also suggests that cognitive impairment in patients with SCI may be associated with inflammatory status (Zhang et al., 2025; Delarue et al., 2025). This means that for remote brain dysfunction after SCI, the evaluation endpoints of terminal protection need to go beyond simple histological improvement and include recovery of functional phenotypes such as cognition and emotion.

On this basis, it is necessary to emphasize that remote brain dysfunction after SCI has clear continuity and stage dependence. Therefore, single-target interventions are unlikely to cover the entire pathological process. In contrast, combined interventions based on disease stage may have higher translational value. In the acute phase, priority may be given to upstream source control, with appropriate consideration of systemic inflammation and BBB protection. In the subacute phase, while continuously restoring intestinal homeostasis, regulation of the immune-inflammatory axis, vagal interface, and stress-related background can be strengthened. In the chronic phase, greater emphasis can be placed on protection of the common downstream brain effector stage, while maintaining long-term management of intestinal homeostasis. It should be noted that this disease-stage framework is mainly a conceptual organization based on the pathological cascade; its purpose is to emphasize that intervention priorities may differ across stages, rather than to propose a fixed treatment regimen that has already been clinically validated.

Based on the above analysis, this review proposes a three-level intervention framework: restoring intestinal homeostasis as the upstream foundation, regulating interorgan relay pathways as the midstream defense, and protecting the common downstream brain effector stage as the downstream protection strategy. The core value of this framework lies in shifting intervention strategies for remote brain dysfunction after SCI from a single-target model to a systematic strategy organized according to the hierarchy of pathological pathways. It also suggests that future treatment models with greater translational potential may need to integrate interventions at different levels according to disease stage and dominant pathological burden.

## 5 Conclusions and future perspectives

Overall, current evidence supports understanding remote brain dysfunction after SCI as a systemic pathological process that extends beyond the injury segment. This review proposes that SCI can gradually generate persistent gut-derived pathological signals through autonomic and neuroendocrine imbalance, intestinal motility disorders, microbial metabolic remodeling, barrier vulnerability, and mucosal immune imbalance. These abnormal signals can then act on the central nervous system through immune-inflammatory, vagal, and neuroendocrine pathways, ultimately converging at a common downstream brain effector stage characterized by activation of neuroinflammatory networks, impaired synaptic plasticity, and dysfunction of the hippocampus and medial prefrontal cortex. From this perspective, cognitive and affective disorders after SCI should not be regarded merely as delayed sequelae of local spinal cord pathology, but may be systemic consequences driven by persistent gut–brain axis dysregulation.

It should be noted that SCI-specific direct evidence fully linking intestinal abnormalities, interorgan transmission, and brain-region dysfunction remains relatively limited. In particular, current understanding of the vagal and neuroendocrine pathways still depends to a large extent on mechanistic integration and evidence from other disease models. Future research should focus on three key directions. First, longitudinal animal models and multi-omics analyses are needed to identify the key causal nodes connecting disruption of intestinal homeostasis with remote brain dysfunction. Second, the dominant mechanisms and optimal intervention windows at different disease stages should be clarified. Third, intervention studies should place greater emphasis on core outcomes such as inflammation in remote brain regions and cognitive and affective phenotypes. Nevertheless, the gut–brain axis framework provides an integrative theoretical basis for re-understanding remote brain dysfunction after SCI. It also suggests that future translational strategies are unlikely to rely only on a single target, but may require a multilevel systematic approach, including restoration of intestinal homeostasis, regulation of interorgan relay pathways, and protection of the common downstream brain effector stage.
